# Experimental Investigation of the Stability of Au_n_Cl_n+m_^−^ (n = 1–5; m = 1, 3, 5, 7) Clusters by Laser Desorption/Ionization Mass Spectrometry

**DOI:** 10.3390/molecules30102227

**Published:** 2025-05-20

**Authors:** Filip Veljković, Xianglei Kong, Stevan Dimitrijević, Marija Janković, Bojan Janković, Vladimir Dodevski, Suzana Veličković

**Affiliations:** 1 ‘‘VINČA” Institute of Nuclear Sciences, National Institute of the Republic of Serbia, University of Belgrade, Mike Petrovica Alasa 12-14, 11351 Belgrade, Serbia; filipveljkovic@vin.bg.ac.rs (F.V.); marijam@vinca.rs (M.J.); bojan.jankovic@vinca.rs (B.J.); vsuzana@vin.bg.ac.rs (S.V.); 2 State Key Laboratory of Elemento-Organic Chemistry, Frontiers Science Center for New Organic Matter, College of Chemistry, Nankai University, Tianjin 300071, China; 3 Tianjin Key Laboratory of Biosensing and Molecular Recognition, College of Chemistry, Nankai University, Tianjin 300071, China; 4 Innovation Centre of Faculty of Technology and Metallurgy Belgrade, University of Belgrade, Karnegijeva 4, 11120 Belgrade, Serbia; sdimitrijevic@tmf.bg.ac.rs

**Keywords:** gold chloride clusters, LDI mass spectrometry, aurophilic interaction, “superhalogen” clusters

## Abstract

The stability of gold chloride clusters is an important topic in catalysis and nanomaterials, but experimental data are missing. Here, fourteen different clusters were obtained simultaneously using laser desorption/ionization mass spectrometry and were identified as Au_n_Cl_n+m_^−^ (n = 1–5; m = 1, 3, 5, 7) or AuCl_n+1_^−^, Au_2_Cl_2n+1_^−^, Au_3_Cl_2n+2_^−^, Au_4_Cl_2n+1_^−^ and Au_5_Cl_2n+2_^−^. Consequently, the effects of laser intensity on their stability were evaluated, considering differences in the AuCl unit or the number of Cl atoms. For the Au_n_Cl_n+1_^−^ and Au_n_Cl_n+3_^−^ groups, the relative intensity of the clusters decreased with each additional AuCl unit as the laser intensity increased. Au_n_Cl_n+5_^−^ clusters showed a different trend in relative intensities: Au_3_Cl_8_^−^ > Au_2_Cl_7_^−^ > Au_4_Cl_9_^−^ > Au_5_Cl_10_^−^. The mononuclear AuCl_4_^−^ showed the highest stability, which is consistent with their “superhalogen” character. In the Au_2_Cl_2n+1_^−^ clusters, Au_2_Cl_5_^−^ with Au (III)–Au(I) interaction was more stable at lower laser intensities, while Au_2_Cl_3_ with Au(I)–Au(I) bonds became more dominant at higher intensities. Among the Au_3_Cl_2n+2_^−^, Au_4_Cl_2n+1_^−^ clusters, those with purely “aurophilic” interactions became increasingly stable with increasing laser intensity. These results emphasize the importance of bond type and cluster size for the stability of gold chloride clusters at different laser intensities.

## 1. Introduction

Understanding the chemical bonding and structure of gold chloride compounds and clusters plays an important role in many fields, such as nanoscience [1,2,3,4,5], environmental science, geological studies [6,7,8,9] and homogeneous and heterogeneous catalysis [10,11,12,13,14,15,16]. Barngrover and colleagues investigated the transformation of gold(III) chloride complexes into gold(I) thiolate species—a key step in the synthesis of thiolate-stabilized gold nanoparticles. Their work highlighted that the ligand exchange between chloride and thiolate ligands occurs with a low-activation energy barrier (~0.35 eV), facilitating the formation of gold chloride–thiolate or gold–thiolate clusters or nanoparticles from gold chloride precursors [2]. In a separate study, Davies and co-workers examined the catalytic behavior of gold supported on carbon (Au/C) in the hydrochlorination of acetylene—an important industrial process for the production of vinyl chloride monomers. Their results demonstrated that Au–Cl complexes play an essential role in catalytic activity [11]. Accordingly, additional insight into the stability of various gold chloride clusters can offer valuable information for understanding nanoparticle formation mechanisms and for designing catalysts with tailored properties.

In general, the chemistry of gold chloride differs from that of other related metals. Metal chloride complexes have mainly ionic properties, whereas gold-containing compounds have a pronounced covalent character. These differences are due to the strong relativistic effect that occurs in gold, leading to the stabilization of the outer 6s orbital and destabilization of the 5d orbitals, resulting in a decrease in the 6s–5d energy gap and an increase in s-d hybridization, which is responsible for the presence of covalent bonds between Au and Cl [17,18,19]. Gold chloride clusters also have an interesting structure and properties [20,21,22,23,24]. Theoretical studies have shown that the anions of the Au_n_Cl_n+1_ cluster (n = 2–7), as the most stable isomers, have a planar zigzag structure characterized by an interesting interaction between the gold atoms. At the base of the cluster structure Au_n_Cl_n+1_ for n > 2, there is a terminal Au···Au interaction (interaction between the terminal gold atom and the neighboring gold atom) and the interaction between two inner neighboring gold atoms (so-called non-terminal Au···Au interaction) [22]. These distances between the terminal and non-terminal gold atom are shorter than the sum of the van der Waals radii of two gold atoms, which is unexpected since the gold(I) centers have a closed-shell electronic configuration [5d^10^]. Therefore, this Au···Au interaction, whose energy lies between the van der Waals and covalent bonding, has been termed the “aurophilic interaction” [25]. An increase in n in Au_n_Cl_n+1_ clusters leads to a stronger “aurophilic interaction” between neighboring gold atoms. It has also been shown that the “aurophilic interactions” in gold–iodine clusters (Au_n_I_n+1_) are stronger than in these gold–chlorine clusters [26]. The distances of Au···Au in the clusters of Au_n_Cl_n+3_ (n = 3–7) are somewhat shorter than the corresponding distances in Au_n_Cl_n+1_. The main structural difference between these clusters is the presence of the AuCl_4_ unit, i.e., there is an Au (III) atom at the edge connected to four Cl atoms, which is not the case in the Au_n_Cl_n+1_ cluster [21,22].

The relativistic effects can be decisive for the stability of the oxidation states of the 5d elements [27]. While the halogen chemistry of silver and copper is restricted to the oxidation states +I and +II, the oxidation states +I and +III are known for gold, while the divalent Au(II) occurs less frequently [28]. Gold can have different oxidation states in anionic, neutral and cationic complexes such as AuCl_2_ (the valence of Au is +I in AuCl_2_^−^, the valence of Au is +II for AuCl_2_ and the valence of Au is +III for AuCl_2_^+^). Since the oxidation states of Au can be increased by adding electronegative ligands such as chlorine (e.g., AuCl, AuCl_2_ and AuCl_3_), AuCl_n_-type gold clusters (n = 1–6) were selected as a good prototype for exploring the maximum possible oxidation states of gold [24,29,30]. Theoretical studies have shown that the participation of d electrons in the bonding increases with the increase in Cl atoms, so +V is the highest possible oxidation state of Au in the AuCl_n_ clusters. It is also shown that with an increase in the number of Cl atoms above 3 in the AuCl_n_ clusters (n= 2–6), a delocalization of the additional electron over several Cl atoms occurs. The consequence of this electron delocalization is the fact that AuCl_n_ clusters (n ≥ 2) have an adiabatic electron affinity (EA) that is greater than the EA of Cl, so these clusters belong to the group of “superhalogens” [31,32,33]. It is interesting to note that the electron affinity of Au_2n_ clusters increases with the addition of Cl atoms. It should be emphasized that in the group of Au_2n_Cl clusters (n = 1−4), the electron affinity of Au_2_Cl is higher than that of Cl, which is very unusual for a multimetal cluster belonging to a group of “superhalogens” [24].

Experimentally, gold chloride clusters are generally obtained in the gas phase using various types of mass spectrometers. Karataev et al., for example, used a high-resolution time-of-flight mass spectrometer with electron impact ionization (EI-TOF-MS) for their experiment [34]. A sample of HAuCl_4_ (weighing 1–2 mg) was placed in a quartz crucible and heated at 100–120 °C in a tantalum furnace. In this way, the following gold clusters were recorded in the positive-mode mass spectrum: Au_2_Cl^+^, Au_2_Cl_2_^+^, Au_2_Cl_3_^+^, Au_2_Cl_4_^+^, Au_2_Cl_6_^+^. Lemke et al. have presented that the mononuclear clusters of the types [AuCl_2_]^+^(H_2_O)_n_ (n = 0–4), [AuOHCl]^+^ (H_2_O)_n_ (n = 0–1) and [AuCl_2_]^+^(HCl)_2_(H_2_O)_n_ (n = 0–4) and the dinuclear [Au_2_Cl_5-x_OH_x_]^+^(H_2_O)_n_ (x = 0–1) can be obtained by electrospray ionization Fourier transform ion cyclotron resonance mass spectrometry (ESI-FTICR-MS). The sample was an aqueous AuCl_3_ solution (concentration 5–50 mM). The results showed that with increasing AuCl_3_ concentration, the abundance of the dinuclear gold–chloride cluster fraction increases, especially [Au_2_Cl_5_]^+^(H_2_O)_n_ [35]. Ma et al. used a matrix-assisted laser desorption/ionization Fourier transform ion cyclotron resonance mass spectrometer (MALDI-FTICR-MS) in their study [23]. The sample was HAuCl_4_ at a concentration of 2 mg/mL prepared in water, while the graphene was a matrix at a concentration of 1 mg/mL dispersed in acetone. First, 1 μL of the graphene dispersion was applied to the stainless target spot, and after drying, 1 μL of the HAuCl_4_ solution was added to the same spot. The mass spectrum was generated using a Nd:YAG laser with typical laser energy of 3 mJ/pulse and a wavelength of 355 nm. In contrast to the two previous cases, the mass spectrum here was recorded in negative ion mode. In this mass spectrum, three main peaks corresponding to Au_3_Cl_2_^−^ > Au_4_Cl^−^ > Au_2_Cl_3_^−^ clusters and low-intensity peaks of Au_5_^−^, Au_3_^−^, Au_7_^−^~Au_6_Cl^−^, Au_8_Cl^−^, Au_5_Cl_2_^−^ and Au_4_Cl_3_^−^ were detected. The researchers in the same group have shown that when the HAuCl_4_ concentration was increased to 20 mg/mL and the laser energy was increased by 10%, the cluster anions Au_3_Cl_4_^−^ > Au_2_Cl_3_^−^ > Au_4_Cl_5_^−^ could be identified. In this case, the cluster anions Au_n_Cl_n−1_^−^ and Au_n_Cl_n_H^−^ were also found with low intensity [22].

It is worth noting that previous studies reported only selected gold chloride clusters with low intensity and insufficient stability for detailed analysis, which made systematic classification difficult. Although previous work showed the detection of Au_n_Cl_n+1_^−^, Au_n_Cl_n+3_^−^ and Au_n_Cl_n+5_^−^, (n = 2–4) by LDI-TOF-MS without graphene, the focus was mainly on theoretical considerations [21]. In contrast, this study provides the first experimental insight into how cluster size—by the sequential addition of AuCl or Cl units—affects stability. By varying the laser intensity, we systematically investigated the relative stabilities of the simultaneously formed clusters. These results provide valuable insights into the stability and transformation of gold chloride clusters and help researchers evaluate their role in relevant systems. The results were also compared with previous mass spectra to assess the influence of graphene and with theoretical data to better understand the stability of clusters.

## 2. Results and Discussion

**Figure 1:**
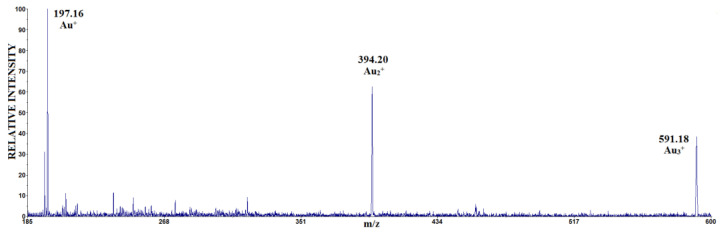
The LDI mass spectrum in the positive mode of HAuCl_4_.

A typical LDI mass spectrum of HAuCl_4_ in the positive mode is shown in Figure 1.

**Figure 2:**
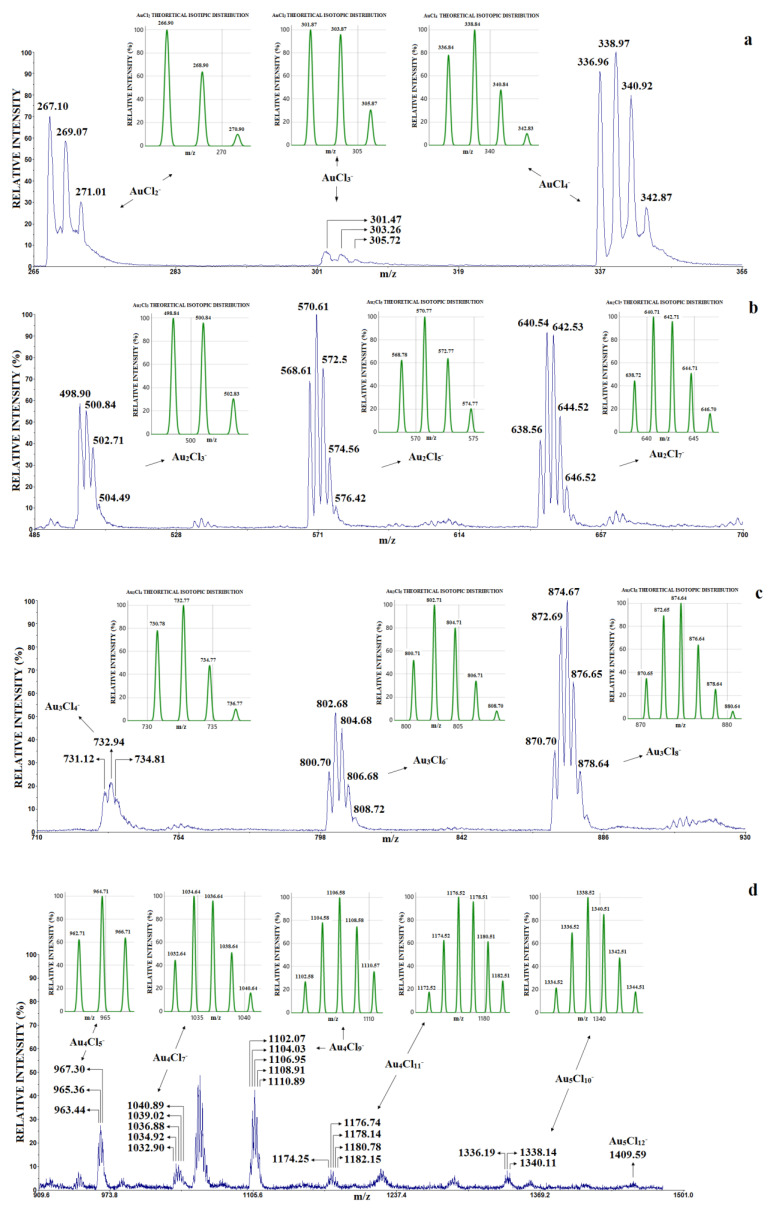
The LDI mass spectrum in the negative mode of HAuCl_4_: (**a**) *m*/*z* 260–360, AuCl_n+1_^−^ (n = 1, 2, 3); (**b**) *m*/*z* 480–700, Au_2_Cl_2n+1_^−^; (**c**) *m*/*z* 700–930, Au_3_Cl_2n+2_^−^; (**d**) *m*/*z* 930–1500, Au_4_Cl_2n+1_^−^ and Au_5_Cl_2n+2_^−^ clusters.

For clarity, the representative LDI mass spectrum of HAuCl_4_ in the negative mode is divided into four parts—*m*/*z* 260–360, *m*/*z* 480–700, *m*/*z* 700–930 and *m*/*z* 930–1500—and shown in Figure 2. The theoretical isotopology of the assumed stoichiometry of the gold chloride clusters is shown in Figure 2 next to the corresponding peak groups.

For an easier comparison of the results obtained in this work with the corresponding results available in the literature, Table 1 lists the ions obtained by other authors from the EI-TOF-MS and MALDI-FTICR mass spectra of HAuCl_4_. It should be noted that Table 1 does not include the ESI-FTICR-MS [35] and LDI-MS results (at a concentration of 2.0–0.025 mg/mL) for HAuCl_4_ [36] because both studies detected hydrated gold chloride clusters, which are not the subject of this study.

In this study, the positive mode of the LDI mass spectrum of HAuCl_4_ contains three peaks at *m*/*z* 197, 394 and 591, which are identified as Au^+^, Au_2_^+^ and Au_3_^+^, respectively (Figure 1). In the laser energy range of 1100–2000 a.u., the individual intensities of the Au_n_^+^ clusters (n = 1, 2, 3) decreased with increasing laser energy, while the ratio of their relative intensities was Au^+^ > Au_2_^+^ > Au_3_^+^.

The comparison of the results in Figure 1 with the results from the literature (Table 1) shows that the choice of ionization method influences the type of clusters detected. For example, the LDI-MS method in positive mode favors the formation of Au_n_^+^ clusters (n = 2, 3), while EI-TOF-MS favors the formation of Au_n_Cl^+^ and Au_2_Cl_n+1_^+^ cations from the same sample [34].

In the negative mode of the LDI mass spectrum of HAuCl_4_ (Figure 2), the following clusters were identified: a “superhalogen” mononuclear cluster of type AuCl_n+1_^−^ (n = 1, 2, 3) in the first part of Figure 2a (*m*/*z* 260–360); a dinuclear cluster of type Au_2_Cl_2n+1_^−^ in the second part of Figure 2b (*m*/*z* 480–700); a trinuclear cluster of type Au_3_Cl_2n+2_^−^ in the third part of Figure 2c (*m*/*z* 700–930); and a tetranuclear cluster of type Au_4_Cl_2n+1_^−^ and pentanuclear Au_5_Cl_2n+2_^−^ clusters in the fourth part of Figure 2d (*m*/*z* 930–1500). These clusters were detected in the laser intensity range from 1200 to 2600 a.u.

Comparing the available results for the positive and negative modes from Table 1, it can be seen that mononuclear and dinuclear clusters can be obtained in the positive-mode EI-TOF-MS [34], while mononuclear, dinuclear, trinuclear and tetranuclear gold chloride clusters were detected in the negative-mode LDI-TOF-MS. It should also be noted that clusters with two gold atoms and an even number of Cl atoms (Au_2_Cl_4_^+^, Au_2_Cl_6_^+^) were detected in the positive EI-TOF-MS mode, while dinuclear gold clusters with an odd number of Cl atoms (Au_2_Cl_5_^−^, Au_2_Cl_7_^−^) are stable in the negative LDI-TOF-MS mode. Interestingly, the dinuclear Au_2_Cl_3_ cluster is stable in both positive and negative modes.

The influence of graphene on the type of the gold chloride clusters can be observed by comparing the results of the LDI and MALDI methods in negative mode (Figure 2 and Table 1). With the MALDI method, clusters of type Au_n_Cl (n = 2, 4, 6, 8), Au_n_ (n = 1–9) and Au_n_Cl_n−1_^−^ (n = 3, 4) were detected (the matrix was graphene and the concentration of HAuCl_4_ was 2 mg/mL, Table 1). The mentioned clusters were not found in the LDI mass spectrum (without graphene and the concentration of HAuCl_4_ was 2.5 mg/mL, Figure 2).

On the other hand, as mentioned above, the anions Au_n_Cl_n+1_, Au_n_Cl_n+3_ and Au_n_Cl_n+5_ were detected in the LDI mass spectrum (without graphene, the concentration of HAuCl_4_ was 2.5 mg/mL, Table 1 and Figure 2). However, Au_n_Cl_n+1_^−^, Au_n_Cl_n−1_^−^, Au_n_Cl_n_H^−^ anions (n = 1–4) were detected in the MALDI mass spectrum and at a much higher concentration (the concentration of HAuCl_4_ was 20 mg/mL, Table 1) than in the LDI method.

These indicates that in the negative mode, the presence of graphene is an important factor affecting the stability of some clusters, such as Au_n_^−^ and Au_n_Cl^−^, Au_n_Cl_n_^−^ and AuCl_n+1_^−^, while graphene suppresses the formation of the anions AuCl_n+1_^−^, AuCl_n+3_^−^ and AuCl_n+5_^−^.

To avoid confusion, it must be emphasized that the clusters identified in Figure 2 can be grouped in two ways: as Au_n_Cl_n+m_^−^ (n = 2–5, m = 1, 3, 5, 7), i.e., as clusters of type AuCl_n+1_^−^, AuCl_n+3_^−^ and AuCl_n+5_^−^ and Au_n_Cl_n+7_^−^ (as shown in previous work, which is consistent with previous theoretical results), or as mononuclear, dinuclear, trinuclear, tetranuclear and pentanuclear gold chloride clusters. In accordance with the above, two issues are discussed below: (1) How the increase in Au_n_Cl_n+1_, Au_n_Cl_n+3_ and Au_n_Cl_n+5_ clusters for the AuCl unit affects its stability in the range of laser intensity from 1200 to 2600 a.u.; (2) How the increase in the number of chlorine atoms affects the stability of AuCl_n+1_^−^, Au_2_Cl_2n+1_^−^, Au_3_Cl_2n+2_^−^, Au_4_Cl_2n+1_^−^ and Au_5_Cl_2n+2_^−^ clusters due to the increase in laser intensity.

**Figure 3:**
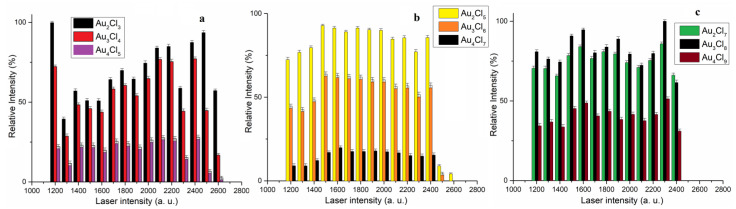
The dependence of the relative intensity of the most abundant isotope of the gold chloride clusters on the laser intensity for (**a**) Au_n_Cl_n+1_^−^ (n = 2–4) clusters; (**b**) Au_n_Cl_n+3_^−^ (n = 2–4); (**c**) Au_n_Cl_n+5_^−^ (n = 2–4) clusters.

Under our experimental conditions, the Au_5_Cl_10_^−^ and Au_5_Cl_12_^−^ (pentanuclear Au_5_Cl_2n+2_ clusters) were of low intensity (Figure 2d) and could therefore not be included in further investigations. The dependence of the relative intensity of the most abundant isotope vs. the laser intensity (in the range of 1200 do 2600 a.u.) for AuCl_n+1_^−^, AuCl_n+3_^−^ and AuCl_n+5_^−^ clusters is presented in Figure 3a, b and c, respectively.

The relative intensity of the Au_n_Cl_n+1_^−^ (n = 2–4) cluster changes in a similar way with increasing laser intensity (Figure 3a). This can be explained by the fact that all clusters of the group Au_n_Cl_n+1_^−^ have similar zigzag structures with the characteristic chemical bond Au(I)-A(I). In this case, the Au_2_Cl_3_^−^ cluster shows the highest stability in the observed range of laser intensity, which is consistent with previous experimental results [22]. Results have shown that increasing the Au_2_Cl_3_ cluster by one AuCl unit leads to a small decrease in stability, and with the addition of another AuCl unit, the stability decreases significantly (Figure 3a). Therefore, the ratio of the relative intensities of Au_n_Cl_n+1_^−^ clusters was Au_2_Cl_3_^−^ > Au_3_Cl_4_^−^ > Au_4_Cl_5_^−^ and does not change with the change in laser intensity. This is consistent with theoretical calculations of their binding energies based on the formula Au_n−1_Cl_n_^−^ + AuCl = AuCl_n+1_, where the binding energies decrease in the series Au_2_Cl_3_ > Au_3_Cl_4_ > Au_4_Cl_5_ [22]. In our experimental conditions, we have shown that the Au_2_Cl_3_ clusters without “aurophilic” interaction are more stable than Au_3_Cl_4_ and Au_4_Cl_5_ clusters with “aurophilic” interaction.

The relative intensity of the Au_n_Cl_n+3_^−^ clusters changes in the same way with increasing laser intensity, as in the previous case (Figure 3b). The most stable cluster in this group is the Au_2_Cl_5_ cluster, which can be described as a fusion of the structural units of AuCl_4_^−^ and AuCl_2_^−^ by the elimination of a Cl atom, without “aurophilic” Au(I)-Au(I) interaction [21]. Also in this case, similar to Au_n_Cl_n+1_^−^, the stability of the cluster decreases with an increasing number of AuCl units, so the Au_4_Cl_7_^−^ cluster has the lowest intensity and could not be detected at laser intensities of 2500 and 2600 a.u. (Figure 3b). In the laser intensity range from 1200 to 2400, the ratio of the relative intensities of the Au_n_Cl_n+3_^−^-type clusters is the same, i.e., Au_2_Cl_5_^−^ > Au_3_Cl_6_^−^ > Au_4_Cl_7_^−^ (Figure 3b).

A comparison of the results in Figure 3a,b shows that increasing the Au_n_Cl_n+3_^−^ cluster by one AuCl unit leads to greater instability than for the Au_n_Cl_n+1_^−^ cluster. It should be noted that for the Au_n_Cl_n+1_^−^ and Au_n_Cl_n+3_^−^ clusters, the “aurophilic” interaction is present when n > 2 [21,22]. Accordingly, the experimental results indicate that the “aurophilic” Au(I)-Au(I) interaction has a more favorable effect on the stability of the Au_n_Cl_n+1_^−^ clusters than the combination of Au(III)-Au(I) and an “aurophilic” Au(I)-Au(I) interaction in the Au_n_Cl_n+3_^−^ cluster.

The Au_n_Cl_n+5_^−^ clusters were detected in a slightly narrower range of laser intensity from 1200 to 2400 (Figure 3c), indicating their lower stability compared to Au_n_Cl_n+1_^−^ and Au_n_Cl_n+3_^−^ (Figure 3a,b). However, it should be noted that the relative intensity of the Au_2_Cl_7_^−^ and Au_3_Cl_8_^−^ clusters was more than 60% in the observed laser intensity ranges. It is interesting to note that four clusters (Au_2_Cl_7_^−^, Au_3_Cl_8_^−^, Au_4_Cl_9_^−^ and Au_5_Cl_10_^−^) were identified in the Au_n_Cl_n+5_^−^ cluster group, in contrast to the previous two groups where three clusters were identified (Figure 2d). The Au_5_Cl_10_^−^ cluster was of low intensity; its relative intensity was between 6 and 10%, but like the other Au_n_Cl_n+5_^−^ clusters, it was detected in the same laser range. In addition, the Au_3_Cl_8_^−^ cluster (the second largest cluster in this group) is more stable than A_2_Cl_7_^−^ and Au_4_Cl_9_^−^. However, it should be noted that the Au_2_Cl^−^ and Au_3_Cl_8_^−^ clusters, which differ from AuCl, do not show a significant difference in relative intensity, as is the case for the Au_n_Cl_n+3_^−^ clusters (Figure 3c). Unfortunately, the results of the theoretical studies on the structure of the Au_n_Cl_n+5_^−^ clusters are missing in the literature, so we cannot compare the structure of the Au_n_Cl_n+5_^−^ clusters with the other two groups.

**Figure 4:**
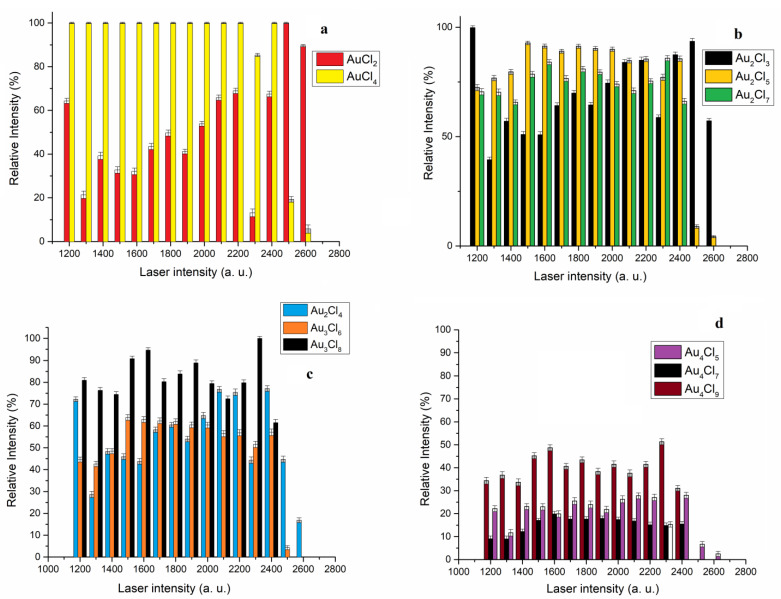
The dependence of the relative intensity of the most abundant isotope of the gold chloride clusters on the laser intensity for (**a**) “superhalogen” mononuclear AuCl_2_^−^ and AuCl_4_^−^ species; (**b**) dinuclear Au_2_Cl_2n+1_^−^ (n = 1, 2, 3) clusters; (**c**) trinuclear Au_3_Cl_2n+2_^−^ (n = 1, 2, 3) clusters; (**d**) tetranuclear Au_4_Cl_2n+1_^−^ (n = 2, 3, 4) clusters.

The dependence of the relative intensity of the most abundant isotope of AuCl_n+1_^−^, Au_2_Cl_2n+1_^−^, Au_3_Cl_2n+2_^−^ and Au_4_Cl_2n+1_^−^ on the laser intensity is shown in Figure 4a, b, c and d, respectively.

The theoretical studies of Srivastava and Misra have shown that the mononuclear AuCl_n_ species (n = 2–6) belong to the group of “superhalogens”, i.e., clusters whose electron affinity is higher than that of Cl (3.6 eV); therefore, it was expected that this type of cluster would dominate in the mass spectrum in the negative mode [24]. Under our experimental conditions, three “superhalogens” of AuCl_n+1_^−^ (n = 1–3) were detected, but the relative intensity of the AuCl_3_^−^ was very low in the observed laser intensity range (Figure 2a). For that reason, the dependence of the relative intensity of the most abundant isotope on the laser intensity for two “superhalogen” (AuCl_4_^−^ and AuCl_2_^−^) is shown in Figure 4a. In the laser intensity range from 1200 to 2400, the dominant anion is AuCl_4_^−^. In this laser intensity range, the relative intensity of AuCl_4_^−^ is significantly higher than the relative intensity of AuCl_2_^−^. This is consistent with the fact that the electron affinity of AuCl_4_ is higher than that of AuCl_2_. However, with increasing laser intensity above 2400 a.u., the dominant ion in the mass spectrum is AuCl_2_^−^. It should be kept in mind that the intensity of the ions in the negative mode is influenced not only by the electron affinity but also by the dissociation energy of the observed clusters. Theoretical studies also show that the dissociation energies of the AuCl_n_ anion for the Cl atom and the Cl_2_ molecule exhibit a similar trend, decreasing in the order AuCl_2_ > AuCl_4_ > AuCl_3_ > AuCl_5_ > AuCl_6_. The dissociation energy for the Cl atom and the Cl_2_ molecule is low for the AuCl_5_, which, despite having the highest EA in the AuCl_n_ series, was not detected in the mass spectrum, and nor was AuCl_6_. The structure of the AuCl_5_ and AuCl_6_ ions is described as (AuCl_4_)Cl_n_ [24]. Theoretical studies by Xu and others have also shown that the structure of the Au_n_Cl_n+3_ cluster is such that it contains an Au(III) ion surrounded by four Cl atoms. Therefore, the AuCl_4_ ion could have been formed by the dissociation of the above clusters. It should be mentioned that the Au_n_Cl_n+1_ clusters have a zigzag structure (without an AuCl_4_ unit), which could be one of the reasons why the AuCl_4_ ion was not detected in the previous work [22]. Regardless of the origin of the “superhalogens” AuCl_2_ and AuCl_4_, their stability under these experimental conditions is more significant than the stability of the other detected clusters.

Theoretical studies have shown that there is no “aurophilic” interaction in the most stable isomers of the dinuclear Au_2_Cl_2n+1_^−^ clusters: Au_2_Cl_3_^−^ and Au_2_Cl_5_^−^. The Au(III)-Au(I) interaction (3.99A) in the Au_2_Cl_5_^−^ cluster ensures that this cluster is more stable than Au_2_Cl_3_^−^ with Au(I)-Au(I) interaction (3.69A) in the laser intensity range from 1300 to 2000 a.u. (Figure 4b). With the increase in laser intensity above 2000 a.u., the intensities of Au_2_Cl_3_^−^ and Au_2_Cl_5_^−^ clusters are very similar, while above 2400 a.u., the Au_2_Cl_3_^−^ cluster is much more stable than Au_2_Cl_5_^−^ and Au_2_Cl_7_^−^. Increasing the number of chlorine atoms in the dinuclear clusters does not significantly affect the stability, so the intensity of the Au_2_Cl_7_ cluster is higher than that of the Au_2_Cl_3_^−^ cluster in the range from 1300 to 1900 and at 2300 a.u., but above 2400 a.u., the Au_2_Cl_7_^−^ cluster is not recorded (Figure 4b).

In the group of Au_3_Cl_n+1_^−^ clusters, the cluster with the largest number of chlorine atoms, Au_3_Cl_8_^−^, shows the highest stability in the range of laser intensity from 1200 a.u. to 2400 a.u.; above 2400 a.u., this cluster was not detected (Figure 4c). The calculated geometrical parameters for the most stable structures available in the literature show that the Au_3_Cl_6_^−^ structure is formed by Au(III)-Au(I) interaction and “aurophilic” Au(I)-Au(I) interaction [21]. This structure is more stable than the structure of the Au_3_Cl_4_^−^ cluster, which is only formed by “aurophilic” interactions in the laser intensity range from 1300 to 2100 a.u. Above 2400 a.u., the intensity of Au_3_Cl_6_^−^ decreases, while the Au_3_Cl_4_^−^ cluster is detected at 2600 a.u.

The tetranuclear Au_4_Cl_2n+1_^−^ clusters exhibit lower stability than the dinuclear and trinuclear clusters in the observed range of laser intensity (Figure 4d). In the group of tetranuclear gold chloride clusters, the largest cluster, Au_4_Cl_9_^−^, is the most stable in the laser intensity range from 1200 to 2400 a.u. In this laser intensity range, the Au_4_Cl_7_^−^ cluster, which contains both Au(III)-Au(I) and thermal and non-thermal “aurophilic” Au(I)-Au(I) bonds, exhibits the lowest stability in the Au_4_Cl_2n+1_^−^ group. The cluster with only thermal and non-thermal “aurophilic” bonds, Au_4_Cl_5_^−^, is between Au_4_Cl_9_^−^ and Au_4_Cl_7_^−^ in intensity. However, it should be noted that the Au_4_Cl_5_^−^ cluster is the only one detected above the laser intensity of 2400 a.u., suggesting that the clusters with only “aurophilic” interactions exhibit greater stability than others with increasing laser intensity.

## 3. Experimental Section

All mass spectra in this work were recorded with a commercially available MALDI TOF mass spectrometer (Voyager-DE PRO Sciex, Foster City, CA, USA). Mass spectra were obtained without the use of conventional or other matrices, i.e., using the laser desorption ionization method. The device was equipped with an ultraviolet N_2_ laser (wavelength 337 nm, pulse width 3 ns and repetition rates of 20.00 Hz). The number of laser shots was 300 per mass spectrum. The instrumental parameters were as follows: An acceleration voltage of 20,000 V, a grating voltage of 94% and a delayed extraction time of 100 ns. These mass spectrometers have a laser attenuator and a prism between the laser and the sample. The laser attenuator is a device for controlling the laser intensity, while the prism deflects the laser beam into the ion source. The laser attenuator is controlled by adjusting the laser level. The laser levels are controlled using the sliders on the manual laser control page. The slider has a range of 0–5000 arbitrary units (a.u.); therefore, this relative scale is used below for the laser intensity. The relative intensity of the cluster anions was determined using five measurements from five different positions. The sample was aqueous HAuCl_4_ solutions (2.5 g/dm^3^) (99%, Sigma-Aldrich, St. Louis, MO, USA) prepared in deionized water (Millipore, Burlington, MA, USA) immediately prior to the experiment. An amount of 0.5 μL of the HAuCl_4_ solution was applied to a spot on the stainless-steel plate. The sample was dried at room temperature and placed in the source area of the mass spectrometer.

## 4. Conclusions

Laser desorption/ionization mass spectrometry is a particularly useful method for the study of gold chloride clusters, as 14 clusters were identified in the LDI mass spectrum of HAuCl_4_, which can be represented in two ways: as Au_n_Cl_n+m-_ (n = 1–5; m = 1, 3, 5, 7) or as AuCl_n+1_^−^-, Au_2_Cl_2n+1_^−^-, Au_3_Cl_2n+2_^−^-, Au_4_Cl_2n+1_^−^- and Au_5_Cl_2n+2_^−^-type clusters. All these clusters were obtained simultaneously, so the effects of increasing the laser intensity on the stability of these clusters could be studied in two cases: when the clusters differed by AuCl unit and when they differed in the number of Cl atoms.

Clusters Au_n_Cl_n+1_^−^ and Au_n_Cl_n+3_^−^ were obtained in the range of laser intensity from 1200 to 2600 a.u., while Au_n_Cl_n+5_^−^ clusters were demonstrated to be less stable than the other two groups of clusters as they were detected in a lower laser intensity range from 1200 to 2400 a.u. With increasing laser intensity, the ratio of the relative intensities of the clusters of groups Au_n_Cl_n+1_^−^ and Au_n_Cl_n+3_^−^ did not change and amounted to Au_2_Cl_3_^−^ > Au_3_Cl_4_^−^ > Au_4_Cl_5_^−^ and Au_2_Cl_5_^−^ > Au_3_Cl_6_^−^ > Au_4_Cl_7_^−^, respectively. This result indicates that increasing the size of clusters Au_n_Cl_n+1_^−^ and Au_n_Cl_n+3_^−^ by one AuCl unit leads to their decreasing stability. Furthermore, increasing the size of the cluster by one AuCl unit has a stronger effect on the stability of the Au_n_Cl_n+3_^−^ cluster than on that of the Au_n_Cl_n+1_^−^ cluster. This indicates that the “aurophilic” Au(I)-Au(I) interaction has a more favorable effect on the stability of the Au_n_Cl_n+1_^−^ cluster than the combination of Au (III)-Au(I) and “aurophilic” Au(I)-Au(I) interaction in the Au_n_Cl_n+3_^−^ cluster. The ratio of the relative intensities of the Au_n_Cl_n+5_^−^ clusters was Au_3_Cl_8_^−^ > A_2_Cl_7_^−^ > Au_4_Cl_9_^−^ > Au_5_Cl_10_^−^, which is different from the previous two cases.

The stability of mononuclear AuCl_4_^−^ and AuCl_2_^−^ species is more significant than that of the other detected clusters. The result is consistent with the theoretically hypothesized “superhalogen” nature of chemical bonding and the calculated dissociation energy for AuCl_4_^−^ and AuCl_2_^−^.

If the gold chloride clusters are classified according to the number of gold atoms into di-, tri-, tetra- and pentanuclear clusters, the Au_2_Cl_3_^−^ and Au_2_Cl_5_^−^ clusters without “aurophilic” interactions exhibit the greatest stability. Au_2_Cl_5_^−^, which is stabilized by an Au(III)-Au(I) interaction, is more stable than Au_2_Cl_3_^−^ up to a laser intensity of 2000 a.u., while Au_2_Cl_3_^−^ (with Au(I)-Au(I) interaction) becomes more stable above 2400 a.u. Among the trinuclear Au_3_Cl_2n+2_^−^ clusters, Au_3_Cl_8_^−^ is the most stable from 1200 to 2400 a.u., while above 2400 a.u., the Au_3_Cl_4_^−^ with “aurophilic” interactions is the most stable. For the tetranuclear Au_4_Cl_2n+1_^−^ clusters, Au_4_Cl_9_^−^ shows the highest stability up to 2400 a.u., but above this intensity, only Au_4_Cl_5_^−^ (with purely “aurophilic” bonds) is detected. These results show that purely “aurophilic” bonds increase the stability of clusters at high laser intensities.

Increasing the number of chlorine atoms has a smaller effect on the stability of gold chloride clusters than increasing the size of the clusters for the AuCl unit.

## Figures and Tables

**Table 1:** Ions identified in previous work using EI-TOF-MS [34] and MALDI- FTICR-MS (at a concentration of 20 mg/mL and 2 mg/mL) of HAuCl_4_ [22,23].

Mass Spectrometry Methods	Ions	Ions	Ions	Ions	Ions
The positive-mode EI-TOF-MS	/	Au_n_Cl^+^ n = 1, 2 AuCl, Au_2_Cl^+^	Au_2_Cl_n+1_^+^ n = 2, 3, 5 Au_2_Cl_3_^+^ Au_2_Cl_4_^+^ Au_2_Cl_6_^+^	/	/
The negative-mode MALDI- FTICR-MS 20 mg/L	/	/	Au_n_Cl_n+1_^−^ n = 1−4 AuCl_2_^−^ Au_2_Cl_3_^−^ Au_3_Cl_4_^−^ Au_4_C_5_^−^	Au_n_Cl_n−1_^−^ n = 2−4 Au_2_Cl^−^ Au_3_Cl_2_^−^ Au_4_Cl_3_^−^	Au_n_Cl_n_H^−^ n = 2−4 Au_2_Cl_2_H^−^ Au_3_Cl_3_^−^
The negative-mode MALDI- FTICR-MS 2 mg/mL	Au_n_^−^ n = 2−9 Au_3_^−^ Au_5_^−^ Au_7_^−^ Au_9_^−^	Au_n_Cl^−^ n = 2, 4, 6, 8 Au_2_Cl^−^ Au_4_Cl^−^ Au_6_Cl^−^ Au_8_Cl^−^	Au_n_Cl_n+1_^−^ n = 2 Au_2_Cl_3_^−^	Au_n_Cl_n−1_^−^ n = 3, 4 Au_3_Cl_2_^−^ Au_4_Cl_3_^−^	/

## Data Availability

The original contributions presented in this study are included in the article. Further inquiries can be directed to the corresponding author.
